# Co-existence of maternal overweight/obesity, child undernutrition, and anaemia among mother-child pairs in Ethiopia

**DOI:** 10.1371/journal.pgph.0002831

**Published:** 2024-03-07

**Authors:** Biniyam Sahiledengle, Lillian Mwanri, Pammla Petrucka, Hiwot Tadesse, Kingsley Emwinyore Agho

**Affiliations:** 1 Department of Public Health, Madda Walabu University Goba Referral Hospital, Bale-Goba, Ethiopia; 2 Research Centre for Public Health Research, Equity and Human Flourishing, Torrens University Australia, Adelaide Campus, Adelaide, South Australia; 3 College of Nursing, University of Saskatchewan, Saskatoon, Canada; 4 Department of Nursing, College of Medicine and Health Sciences, Arba Minch University, Arba Minch, Ethiopia; 5 School of Health Sciences, Western Sydney University, Penrith, NSW, Australia; 6 Translational Health Research Institute, School of Medicine, Western Sydney University, Campbelltown Campus, Penrith, NSW, Australia; 7 Faculty of Health Sciences, University of Johannesburg, Johannesburg, South Africa; PLOS: Public Library of Science, UNITED STATES

## Abstract

Ethiopia is currently known to be the most food-insecure country in sub-Saharan Africa, where childhood undernutrition remains endemic. While attention is increasingly being paid to childhood undernutrition in Ethiopia, a current surge of "triple burden of malnutrition" (TBM) has received less attention. The purpose of this study was to determine the prevalence of TBM and identify the associated factors in Ethiopia. Data were from the Ethiopian Demographic and Health Surveys (2005–2016) and a total of 20,994 mother-child pairs were examined in this study. The TBM was our primary outcome variable, which encompasses three types of nutritional problems-when a mother may be overweight/obese, while her child is stunted, wasted, or underweight plus has anaemia under the same roof. A multilevel logistic regression explored the individual- and community-level factors associated with TBM. Our study indicated that children under-five years of age were anaemic, stunted, wasted, and underweight [49.3% (95% CI: 48.7–49.9), 43.1% (95% CI: 42.4–43.7), 10.3% (95% CI: 9.9–10.7), and 27.6% (95% CI: 27.0–28.1)] respectively. The overall prevalence of TBM was 2.6% (95% CI: 2.39–2.83). Multilevel analyses revealed that TBM was more likely to occur among children aged 12–23 months (AOR: 2.54, 95% CI: 1.68–3.83), 24–35 months (AOR: 1.54, 95% CI: 1.03–2.29), children perceived by their mothers to be smaller than normal at birth (AOR: 1.94, 95% CI: 1.48–2.56), who experienced fever in the past 2 weeks (AOR: 1.58, 95% CI: 1.24–2.01), and lived in urban settings (AOR: 1.79, 95% CI: 1.13–2.86). Lower odds of TBM were reported among female children (AOR: 0.59, 95% CI: 0.47–0.72), and those who lived in rich households (AOR: 0.69: 95% CI: 0.49–0.98). TBM was found to be present in almost three percent of households in Ethiopia. Addressing the TBM through double-duty actions will be of critical importance in achieving malnutrition in all its forms in Ethiopia.

## Introduction

Due to the global nutrition transition, there is a growing concern about the co-occurrence of undernutrition (i.e., wasting, stunting, and underweight) and overnutrition among the members of a single household, a condition termed double burden of malnutrition (DBM) and when accompanied by micronutrient deficiencies leading to anaemia it is termed triple burden of malnutrition (TBM) [1]. The DBM can present at the household level when a mother may be overweight/obese, while a child is categorized as undernourished (i.e., stunting, wasting, or underweight) [2]. The TBM at the household level encompasses co-existance of three types of nutritional problems in a single household, such as a mother being overweight/obese, while her child is stunted, wasted, or underweight plus experiencing anaemia [3]. TBM is a complex problem, which it has not received much attention despite being a potential problem in many low-and middle-income countries (LMICs) where undernutrition remains prevalent [3–6].

The rapid urbanisation and exponential population growth in LMICs have increased the risk of exacerbated undernutrition inequalities, putting pressure on and collapsing ongoing efforts to combat undernutrition [1,7]. Sub‐Saharan Africa (SSA) has been identified as the most affected region with childhood undernutrition [8]. The latest estimate showed that the pooled prevalence of stunting among under-five children in SSA was 35% [9], and six in every ten (60.2%) children aged 6–59 months were affected by anaemia [10]. Of the sub-regions, the highest prevalence of stunting (37%) was in East Africa, where Ethiopia is located [9]. In Ethiopia, the burden of childhood stunting (37%), wasting (21%), and being underweight (7%) have been reported [11], with national prevalence of anemia estimated to be 56% among children under the age of 5 years [12], indicative of a severe public health problem [13].

Few studies have been conducted on TBM in mother-child pairs living under the same roof in LMICs [3,4,6,14]. Previous studies have reported the prevalence of TBM to be 5.7% in India [14], 7.0% in Nepal [3], and 1.2% in Ethiopia [6]. Ahinkorah et al. [4] estimated the prevalence of TBM in SSA to be one percent. The most important contributing factors of TBM among mother-child pairs include the age of the child [4], being a female child [15], perceived size at birth [4,14], age of the mother [14], mothers with short stature [3], mothers who did not attend ANC [4], lower maternal education [3,4,14], large family size/household size [15,16], and time to and from a water source [6], place of residence [14], and wealth status [3,14].

Ethiopia has been committed to reducing the prevalence of all forms of undernutrition [17,18]. Despite efforts, the country is far from meeting the global commitment to achieve meaningful undernutrition reductions. On the other hand, the coexistence of overweight and undernutrition among a single household members is a relatively new concept and has received little attention in the country [6,16,19]. So far, there are limited studies on the TBM at the household level in mother-child dyad in Ethiopia [6,20]. For instance, Tarekegn et al. explored TBM using a single snapshot survey and reported the prevalence at 1.2% (95%CI: 0.83–1.57) [6]. On the other hand, Pradeilles and colleagues explored household-level DBM, defined as the coexistence of maternal overweight/obesity and child undernutrition (i.e., stunting or anaemia) [20]. Beyond these two examples, little is known about TBM in Ethiopia and previous studies have concentrated on the individual-level DBM [16,19,21–24]. To create impactful policies and practices, it is crucial to understand the burden and determinants of TBM. Additionally, comprehensive and timely accounting of the prevalence of TBM is crucial for monitoring progress towards Sustainable Development Goals (SDG) targets. Therefore, the aim of this study is twofold: (i) to assess the prevalence of TBM (i.e., coexistence of overweight/obese plus anaemia in mothers paired with their undernourished child), and (ii) to identify associated factors of TBM in Ethiopia.

## Methods

### The setting, data source, and study design

Data from the 2005, 2011, and 2016 rounds of the Ethiopian Demographic and Health Surveys (EDHS), which comprise data for both anthropometric and anaemia measurements were used in this study. The EDHS uses a stratified two‐stage cluster sampling method and contains nationally and regionally representative cross‐sectional data for households [12,25,26]. In the first stage, after the nine administrative units were stratified into urban and rural strata, Enumeration Areas (EAs) were selected proportional to the household size of the cluster. In the second stage, a fixed number of households per cluster were selected with an equal probability of systematic selection from the newly created household listing. At the time of those consecutive surveys, Ethiopia had nine geographical regions (namely, Afar, Amhara, Benishangul-Gumuz, Gambella, Harari, Oromia, Somali, Southern Nations and Nationalities and People (SNNP), and Tigray) and two administrative cities (i.e., Addis Ababa and Dire Dawa). The complete sampling procedure has been elaborated in the final reports of EDHS [12,25,26]. The EDHS captured childhood anemia for the first time in 2^nd^ survey (EDHS-2005); hence, our analysis was restricted to data from the EDHS (2005–2016). A weighted total of 20,994 mother-child pairs with complete anthropometric and haemoglobin records were considered for this analysis.

### Outcome variable

The outcome variable for the study was the triple burden of malnutrition (TBM) which is defined based on previous similar literature as overweight/obese mothers paired with their child having one form of undernourished (stunted or wasted or underweight) plus anaemia [3,6]. Accordingly, a binary response variable TBM was created and assigned as “1” if an overweight/obese mother was paired with her undernourished plus anaemic child, otherwise coded “0”. The WHO Child Growth Standard classification was used for all anthropometric failures and height-for-age (HAZ), weight-for-height (WHZ), and weight-for-age (WAZ) z-scores below −2 SD were used to define stunting, wasting, and underweight, respectively [27]. Maternal nutritional status was classified as underweight (<18.5 kg/ m^2^), normal (18.5 to < 24.99 kg/m^2^), or overweight/obesity ≥ 25.0 kg/m^2^) [28]. The EDHS collected blood samples among all children of age 6 to 59 months included in the survey for hemoglobin tests. A hemoglobin level of less than 11 grams/deciliter was categorized as anemia as recommended by the World Health Organization (WHO) for classifying anemia [29].

### Independent variables

In this study, the potential confounding variables were based on previous studies [4,6,14,30,31] and their availability in the EDHS dataset. The identified factors were categorized into individual and community-level factors. The individual level variables consisted of sex of the child (male, female), age of the child (6–11 months, 12–23, 24–35, and 36–59 months), birth order (first born, 2–4, or 5 or higher), perceived size of the child at birth (large, average and small), currently breastfeeding (yes, no), experienced diarrhea in the last 2 weeks (yes, no), experienced fever in the last 2 weeks (yes, no), full vaccination (yes, no), received deworming medication in that last 6 month (yes, no), received vitamin A in last 6 months (yes, no), mother’s age (<18, 18–24, 25–34, or ≥35), mother’s education (no education, primary, and above), mother’s occupation (not working, non-agriculture, or agriculture), antenatal care (ANC) visits (none, 1–3, 4–7, and 8 +), maternal stature (normal/tall (≥155 cm), short (145 to 154.9 cm), very short (<145 cm)), wealth index (poor, middle, and rich), household size (1–4, ≥ 5), type of cooking fuel (clean fuels, solid fuels), housing status (built from finished materials, built from natural or unfinished materials), toilet facility (improved, unimproved, open defecation), source of drinking water (improved, unimproved), time to get a water source (on premises, ≤ 30 min round-trip fetching time, 31–60 min round-trip fetching time, and over 60 min round-trip fetching time). Community-level factors included the place of residence (rural, urban) and contextual regions (agrarian, pastoralist, or city administrations).

### Data analysis

All analyses were carried out using STATA version 14 statistical software. The *’Svy’* commands were employed to allow for adjustments for the cluster-sampling design and weight. We conducted frequency tabulations to describe the data used in the study and the distributions of TBM by background characteristics. Due to the hierarchical nature of DHS data (i.e., nested data) a multilevel model was used to assess the association between TBM and individual and community-level determinants. First, multilevel bivariable analysis was performed to assess factors associated with TBM. Variables with a p-value < 0.25 obtained in the multilevel bivariable analysis were selected to enter multilevel multivariable logistic regression models to estimate their independent association with TBM. Subsequently, four models were used. The empty model without any explanatory variables was run to detect the presence of a possible contextual effect (*model I*); the second with individual-level variables (*model II*), the third with community-level variables (*model III*), and the fourth with both individual and community-level variables (*model IV*). Multicollinearity between the potential predictors was checked using a tolerance test. Multicollinearity among independent variables was assessed by using the variance inflation factor (VIF) [32]. Variation between clusters were assessed by computing intra-class correlation coefficient (ICC) [33]. Model comparisons were done using the deviance information criteria (DIC). The model with the lowest DIC was considered the best-fit model. Finally, the fourth model with the lowest information criteria value was chosen as the final best-fit model. Adjusted odds ratio (AOR) with 95% CI was used to determine the strength of the association with a P-value < 0.05 considered as statistically significant.

### Ethics statement

We used datasets provided by the Demographic Health Surveys programme and have not had any form of contact with the study participants. Informed consent for the present analysis was not necessary because secondary data analysis did not involve interaction with the participants. This study was based on an analysis of existing public domain survey datasets that are freely available online with all identifier information removed. Data is publicly available in open access repository and available here: http://dhsprogram.com/. Ethical clearance for the Demographic Health Survey (DHS) was provided by the Ethiopia Health and Nutrition Research Institute (EHNRI) Review Board, the National Research Ethics Review Committee (NRERC) at the Ministry of Science and Technology, the Institutional Review Board of ICF International, and the CDC. The DHS programme recognizes and adheres to established international and local ethical standards and protocols in its surveys. Further information regarding the DHS data usage and ethical standards can be accessed online (https://dhsprogramcom/data/Access-Instructionscfm).

## Results

### Characteristics of the study population

In this analysis, weighted data of 20,994 mother-child pairs were included (EDHS-2005, n = 3,879; EDHS-2011, n = 8,898; EDHS-2016, n = 8,217). Out of these, 48.9% were female, 46% were in the age category of 36–59 months, 70.6% were currently breastfeeding, and 14.5% reported having diarrhea in the last fifty days prior to the survey. Nearly 27.2% of the children were smaller than average size at birth. Only about 50.4% and 14.1% of children received vitamin A and deworming medication in the last 6 months prior to the survey. Most of the mothers were uneducated (70.1%), not working (53.0%), and had no ANC visit during pregnancy (51.2%). A total of 45.4% of mother-child dyads were from the relatively poor wealth category, while almost 10% of households had improved toilet facilities and 45.8% used improved sources of drinking water (**Table 1**).

**Table 1:** Socio‐demographic characteristics of the sample population and prevalence of mother-child pairs of triple burden of malnutrition by characteristics of the study population, EDHS (2005–2016).

Variables	Weighted, (n)	Weighted, (%)
***Individual-level characteristics***		
***Child factors***		
**Sex**		
Male	10,723	51.1
Female	10,270	48.9
**Age (months)**		
6–11	2,360	11.3
12–23	4,510	21.6
24–35	4,426	21.1
36–59	9,636	46.0
**Birth order**		
Firstborn	3,673	17.5
2–4	9,127	43.5
5 or higher	8,193	39.0
**Birth interval**		
7–33 months	14,441	68.8
≥33 months	6,553	31.2
**Size of a child at birth**		
Larger	6,747	32.2
Average	8,494	40.6
Small	5,686	27.2
**Currently breastfeeding**		
Yes	14,825	70.6
No	6,169	29.4
**Full vaccination**		
Yes	4,132	24.9
No	12,454	75.1
**Diarrhea**		
Yes	3,033	14.5
No	17,930	85.5
**Fever**		
Yes	3,534	16.9
No	17,419	83.1
**Deworming medication in the last 6 months**		
Yes	2,949	14.1
No	18,044	85.9
**Vitamin A in last 6 months**		
Yes	10,393	50.4
No	10,220	49.6
***Parental factors***		
**Mother’s age**		
15–18	103	0.5
18–24	4,498	21.4
25–34	11.042	52.6
≥35	5,351	25.5
**Mother’s education**		
No education	14,723	70.1
Primary and above	6,270	29.9
**Mother’s occupation**		
Not working	11,086	53.0
Non agriculture	4,618	22.1
Agriculture	5,202	24.9
**Antenatal care (ANC) visit(s)**		
None	6,991	51.2
1–3	3,423	25.1
4–7	2,912	21.3
8+	315	2.3
**Maternal stature**		
Normal/Tall (≥155 cm)	13,003	61.9
Short (145 to 154.9 cm)	7,492	35.7
Very short (<145 cm)	499	2.4
**Listening to radio**		
Yes	7,952	37.9
Not at all	13,033	62.1
**Watching television**		
Yes	4,612	22.0
Not at all	16,369	78.0
***Household factors***		
**Wealth index**		
Poor	9,531	45.4
Middle	4,409	21.0
Rich	7,053	33.6
**Household size**		
1–4	4,965	23.6
≥ 5	16,028	76.4
**Type of cooking fuel**		
Clean fuels	289	1.4
Solid fuels	20,449	98.6
**Housing status**		
Built from finished materials	438	2.1
Built from natural or unfinished materials	20,548	97.9
**Toilet facility**		
Improved	2,111	10.2
Unimproved	9,277	44.7
Open defecation	9,350	45.1
**Source of drinking water**		
Improved	9,493	45.8
Unimproved	11,239	54.2
**Child stool disposal**		
Safe	5,578	26.7
Unsafe	15,326	73.3
**Time to get a water source**		
On-premise	1,478	7.0
≤ 30 min	11,751	55.9
31–60 min	4,483	21.3
>60 min	3,280	15.6
***Community-level characteristics***		
**Residence**		
Urban	2,269	10.8
Rural	18,724	89.2
**Region**		
Agrarian	11,416	54.4
Pastoralist	9,087	43.3
City administration	490	2.3
**Survey year**		
EDHS-2005	3,879	18.5
EDHS-2011	8,898	42.4
EDHS-2016	8,217	39.1

### Prevalence of TBM

**Fig 1:**
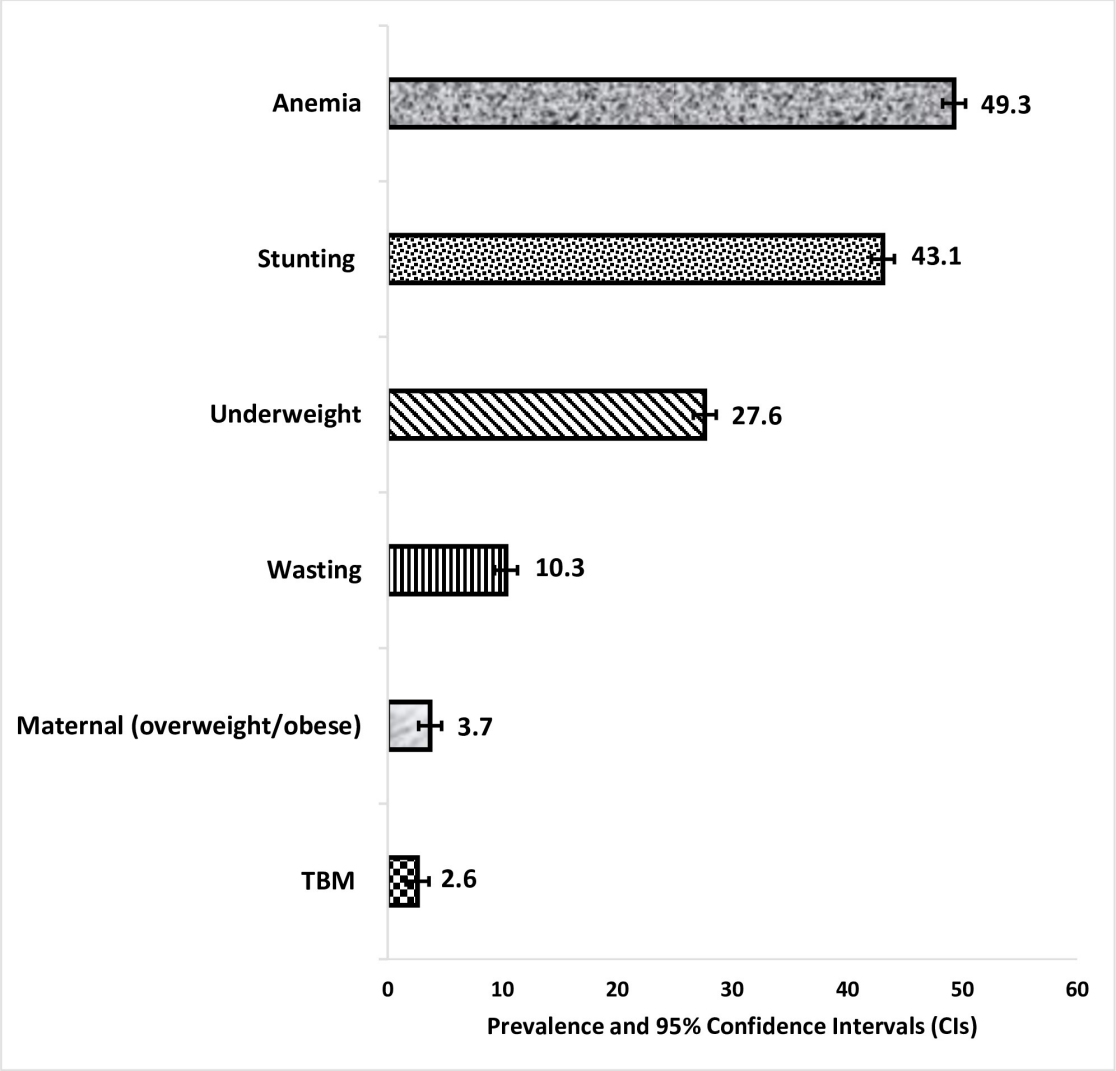
Prevalence of malnutrition and TBM in Ethiopia (EDHS 2005–2016).

**Fig 1** represents the percentage of the nutritional status of mothers and children in Ethiopia. It was found that about 43.1% (95% CI: 42.4–43.7), 10.3% (95% CI: 9.9–10.7), and 27.6% (95% CI: 27.0–28.1) were stunted, wasted and underweight, respectively. Almost half, 49.3% (95% CI: 48.7–49.9) of children were anemic. It was also found that 3.7% (95% CI: 3.50–3.96) of women were overweight/obese.

The overall prevalence of TBM was 2.6% (95% CI: 2.39–2.83). The prevalence of TBM dropped from 3.6% (95% CI: 3.06–4.24) from the EDHS-2005 to 2.4% (95% CI: 2.12–2.76) in EDHS-2011 and 2.3% (95% CI: 2.01–2.66) in EDHS-2016 (**Table 2**).

**Table 2:** Unadjusted association between the triple burden of malnutrition (TBM) and maternal heights and other study covariates among mother-child pairs in Ethiopia, EDHS (2005–2016).

Variables	Prevalence of TBM, 95%CI	Unadjusted OR (95%CI)	p-value
***Individual-level characteristics***			
***Child factors***			
**Sex**			
Male	3.72 (3.37–4.12)	Ref.	
Female	2.55 (2.25–2.89)	0.67 (0.57–0.79)	p<0.001
**Age (months)**			
6–11	2.28 (2.23–3.64)	1.56 (1.16–2.09)	0.003
12–23	5.81 (5.14–6.55)	3.26 (2.66–3.99)	p<0.001
24–35	3.34 (2.83–3.93)	1.82 (1.45–2.29)	p<0.001
36–59	1.87 (1.61–2.18)	Ref.	
**Birth order**			
Firstborn	2.19 (1.76–2.72)	0.57 (0.45–0.74)	p<0.001
2–4	3.08 (2.74–3.47)	0.81 (0.68–0.97)	0.023
5 or higher	3.72 (3.30–4.18)	Ref.	
**Birth interval**			
7–33 months	3.16 (2.88–3.47)	Ref.	
≥33 months	3.12 (2.71–3.59)	0.99 (0.83–1.18)	0.924
**Size of a child at birth**			
Larger	2.01 (1.67–2.39)	Ref.	
Average	2.80 (2.46–3.19)	1.40 (1.12–1.76)	0.003
Small	4.89 (4.35–5.49)	2.49 (2.00–3.12)	p<0.001
**Currently breastfeeding**			
Yes	3.45 (3.15–3.78)	Ref.	
No	2.54 (2.18–2.95)	0.72 (0.59–0.86)	p<0.001
**Full vaccination**			
Yes	2.36 (1.95–2.86)	Ref.	
No	3.82 (3.48–4.19)	1.63 (1.31–2.04)	p<0.001
**Diarrhea**			
Yes	4.88 (4.16–5.73)	1.79 (1.47–2.18)	p<0.001
No	2.84 (2.60–3.11)	Ref.	
**Fever**			
Yes	5.31 (4.61–6.11)	2.06 (1.72–2.47)	p<0.001
No	2.68 (2.44–2.94)	Ref.	
**Deworming medication in the last 6 months**			
Yes	2.50 (1.98–3.15)	Ref.	
No	3.25 (2.99–3.53)	1.30 (1.01–1.68)	0.042
**Vitamin A in last 6 months**			
Yes	50.4	Ref.	
No	49.6	1.37 (1.59–1.61)	p<0.001
***Parental factors***			
**Mother’s age**			
<18	3.06 (0.98–9.11)	0.89 (0.27–2.87)	0.850
18–24	3.32 (2.83–3.90)	0.99 (0.79–1.25)	0.983
25–34	2.99 (2.68–3.34)	0.89 (0.73–1.08)	0.248
≥35	3.32 (2.85–3.87)	Ref.	
**Mother’s education**			
No education	3.70 (3.39–4.03)	1.96 (1.59–2.42)	p<0.001
Primary and above	1.91 (1.59–2.29)	Ref.	
**Mother’s occupation**			
Not working	3.64 (3.31–4.01)	Ref.	
Non agriculture	1.84 (1.47–2.29)	0.49 (0.38–0.64)	p<0.001
Agriculture	3.13 (2.64–3.72)	0.87 (0.71–1.07)	0.192
**Antenatal care (ANC) visit**			
None	5.24 (4.71–5.83)	Ref.	
1–3	3.09 (2.53–3.76)	0.57 (0.45–0.72)	p<0.001
4–7	1.77 (1.36–2.31)	0.32 (0.24–0.43)	p<0.001
8+	0.61 (0.19–1.88)	0.11 (0.03–0.34)	p<0.001
**Maternal stature**			
Normal/Tall (≥ 155 cm)	2.91 (2.63–3.22)	Ref.	
Short (145 to 154.9 cm)	3.58 (3.15–4.07)	1.23 (1.04–1.46)	0.015
Very short (<145 cm)	3.84 (2.33–6.28)	1.34 (0.78–2.28)	0.281
**Listening to radio**			
Yes	1.98 (1.67–2.34)	Ref.	
Not at all	3.75 (3.43–4.09)	1.92 (1.57–2.33)	p<0.001
**Watching television**			
Yes	1.54 (1.22–1.95)	Ref.	
Not at all	3.62 (3.33–3.93)	2.39 (1.85–3.09)	p<0.001
***Household factors***			
**Wealth index**			
Poor	4.27 (3.88–4.69)	Ref.	
Middle	2.84 (2.31–3.48)	0.66 (0.52–0.83)	0.001
Rich	1.63 (1.35–1.97)	0.36 (0.29–0.45)	p<0.001
**Household size**			
1–4	2.64 (2.22–3.13)	0.78 (0.64–0.96)	0.019
≥ 5	3.32 (3.04–3.62)	Ref.	
**Housing status**			
Built from finished materials	0.09 (0.48–1.77)	Ref.	
Built from natural or unfinished materials	3.26 (3.02–3.53)	3.70 (1.90–7.20)	p<0.001
**Toilet facility**			
Improved	1.72 (1.31–2.27)	Ref.	
Unimproved	2.36 (2.02–2.76)	1.42 (1.03–1.97)	0.033
Open defecation	4.07 (3.69–4.47)	2.48 (1.83–3.35)	p<0.001
**Source of drinking water**			
Improved	2.77 (2.44–3.15)	Ref.	
Unimproved	3.41 (3.08–3.77)	1.22 (1.03–1.45)	0.019
**Child stool disposal**			
Safe	2.16 (1.79–2.60)	Ref.	
Unsafe	3.51 (3.21–3.82)	1.63 (1.32–2.02)	p<0.001
**Time to get a water source**			
On-premise	1.22 (0.83–1.78)	Ref.	
≤ 30 min	2.76 (2.46–3.11)	2.32 (1.54–3.49)	p<0.001
31–60 min	3.80 (3.23–4.46)	3.26 (2.12–4.99)	p<0.001
>60 min	4.55 (3.94–5.26)	3.88 (2.55–5.92)	p<0.001
***Community-level characteristics***			
**Residence**			
Urban	1.76 (1.36–2.29)	0.49 (0.37–0.65)	p<0.001
Rural	3.42 (3.15–3.71)	Ref.	
**Region**			
Agrarian	3.17 (2.87–3.51)	Ref.	
Pastoralist	3.87 (3.36–4.45)	1.22 (1.01–1.48)	0.036
City administration	1.74 (1.32–2.31)	0.51 (0.37–0.70)	p<0.001
**Survey year**			
EDHS-2005	3.38 (2.82–4.03)	1.15 (0.92–1.45)	0.219
EDHS-2011	3.18 (2.83–3.57)	1.07 (0.89–1.28)	0.492
EDHS-2016	3.00 (2.64–3.41)	Ref.	

### Factors associated with TBM

**Tables 2** and **3** present results for the bivariate and multivariable multilevel associations of background characteristics with TBM among mother-child pairs in Ethiopia, respectively. Bivariate association indicates that TBM was associated with child, maternal, and household factors (**Table 2**). TBM was less likely to occur among female children compared to males (AOR: 0.59, 95% CI: 0.47–0.72). Children aged 12–23 months (AOR: 2.54, 95% CI: 1.68–3.83) and aged 24–35 months (AOR: 1.54, 95% CI: 1.03–2.29) were more likely to experience TBM compared to those aged 36–59 months. Children perceived smaller for size than average at birth were more likely to suffer from TBM compared to those who were larger than average at birth (AOR: 1.94, 95% CI: 1.48–2.56). The odds of TBM were higher among children who had a fever in the last fifty days prior to the survey (AOR: 1.58, 95% CI: 1.24–2.01) than their counterparts. Mothers with a non-agriculture occupation (AOR: 0.64, 95% CI: 0.46–0.88), mothers who had one to three antenatal care (ANC) visits (AOR: 0.73, 95% CI: 0.56–0.96) and 4–7 ANC visits (AOR: 95% CI: 0.50, 0.34–0.72) were lower odds of experiencing TBM. Children born to mothers with short height (AOR: 1.43, 95% CI: 1.15–1.76) had higher odds of experiencing TBM compared to those born to mothers who had normal height. TBM was less likely to occur among households within the relatively rich wealth category compared to poor (AOR: 0.69: 95% CI: 0.49–0.98). Children born to households that spent a long-time fetching water had higher odds of TBM than children born to households that had drinking water on their premises. The odds of TBM were more likely to occur among urban residents compared to rural (AOR: 1.79, 95% CI: 1.13–2.86) (**Table 3**).

**Table 3:** Adjusted association between prevalence of mother-child pairs of triple burden of malnutrition by characteristics of the study population, EDHS (2005–2016).

Variables	Model 1	Model 2	Mode 3	Model 4
	Null model	AOR (95%CI)	AOR (95%CI)	AOR (95%CI)
***Individual-level characteristics***				
***Child factors***				
**Sex**				
Male		Ref.		Ref.
Female		0.59 (0.47–0.72)**		0.59 (0.47–0.72)**
**Age (months)**				
6–11		1.10 (0.68–1.77)		1.07 (0.66–1.74)
12–23		2.60 (1.74–3.88)**		2.54 (1.68–3.83)**
24–35		1.58 (1.06–2.34)*		1.54 (1.03–2.29)*
36–59		Ref.		Ref.
**Birth order**				
Firstborn		0.74 (0.47–1.17)		0.73 (0.46–1.16)
2–4		0.91 (0.69–1.19)		0.91 (0.69–1.19)
5 or higher		Ref.		Ref.
**Size of a child at birth**				
Larger		Ref.		Ref.
Average		1.31 (0.99–1.72)		1.30 (0.98–1.72)
Small		1.94 (1.47–2.54)**		1.94 (1.48–2.56)*
**Currently breastfeeding**				
Yes		Ref.		Ref.
No		0.90 (0.68–1.18)		0.89 (0.68–1.18)
**Full vaccination**				
Yes		Ref.		Ref.
No		1.11 (0.84–1.45)		1.13 (0.86–1.49)
**Diarrhea**				
Yes		1.26 (0.98–1.61)		1.27 (0.99–1.63)
No		Ref.		Ref.
**Fever**				
Yes		1.57 (1.23–1.99)**		1.58 (1.24–2.01)**
No		Ref.		Ref.
**Deworming medication in the last 6 months**				
Yes		Ref.		Ref.
No		0.95 (0.67–1.33)		0.93 (0.65–1.32)
**Vitamin A in last 6 months**				
Yes		Ref.		Ref.
No		1.14 (0.92–1.42)		1.14 (0.91–1.41)
***Parental factors***				
**Mother’s age**				
<18		0.76 (0.17–3.43)		0.79 (0.17–3.58)
18–24		1.16 (0.78–1.73)		1.17 (0.78–1.75)
25–34		1.01 (0.76–1.31)		0.99 (0.76–1.31)
≥35		Ref.		Ref.
**Mother’s education**				
No education		1.17 (0.88–1.55)		1.20 (0.90–1.59)
Primary and above		Ref.		Ref.
**Mother’s occupation**				
Not working		Ref.		Ref.
Non-agriculture		0.65 (0.47–0.89)*		0.64 (0.46–0.88)*
Agriculture		0.90 (0.69–1.18)		0.92 (0.70–1.20)
**Antenatal care (ANC) visit**				
None		Ref.		Ref.
1–3		0.76 (0.58–0.99)*		0.73 (0.56–0.96)*
4–7		0.54 (0.38–0.76)*		0.50 (0.34–0.72)**
8+		0.39 (0.12–1.29)		0.35 (0.11–1.18)
**Maternal stature**				
Normal/Tall (≥155 cm)		Ref.		Ref.
Short (145 to 154.9 cm)		1.41 (1.14–1.74)*		1.43 (1.15–1.76)*
Very short (<145 cm)		1.68 (0.90–3.12)		1.69 (0.91–3.15)
**Listening to radio**				
Yes		Ref.		Ref.
Not at all		1.35 (1.04–1.74)*		1.32 (0.79–1.62)
**Watching television**				
Yes		Ref.		Ref.
Not at all		1.01 (0.77–1.56)		1.14 (0.79–1.63)
***Household factors***				
**Wealth index**				
Poor		Ref.		Ref.
Middle		0.79 (0.58–1.08)		0.81 (0.59–1.11)
Rich		0.75 (0.54–1.04)		0.69 (0.49–0.98)*
**Household size**				
1–4		0.82 (0.62–1.09)		0.81 (0.61–1.08)
≥ 5		Ref.		Ref.
**Housing status**				
Built from finished materials		Ref.		Ref.
Built from natural or unfinished materials		1.42 (0.54–3.71)		1.62 (0.61–4.27)
**Toilet facility**				
Improved		Ref.		Ref.
Unimproved		1.10 (0.70–1.72)		1.17 (0.74–1.83)
Open defecation		1.28 (0.82–2.03)		1.36 (0.85–2.15)
**Source of drinking water**				
Improved		Ref.		Ref.
Unimproved		0.83 (0.67–1.04)		0.86 (0.67–1.09)
**Child stool disposal**				
Safe		Ref.		Ref.
Unsafe		1.02 (0.76–1.37)		1.04 (0.77–1.40)
**Time to get a water source**				
On-premise		Ref.		Ref.
≤ 30 min		1.55 (0.84–2.85)		1.89 (1.01–3.57)*
31–60 min		1.81 (0.95–3.40)		2.22 (1.15–4.28)*
>60 min		1.85 (0.98–3.50)		2.30 (1.19–4.46)*
***Community-level characteristics***				
**Residence**				
Urban			0.58 (0.43–0.79)*	1.79 (1.13–2.86)*
Rural			Ref.	Ref.
**Region**				
Agrarian			Ref.	Ref.
Pastoralist			1.20 (0.99–1.46)	0.96 (0.74–1.24)
City administration			0.62 (0.44–0.86)*	0.94 (0.62–1.42)
**Survey year**				
EDHS-2005			1.14 (0.90–1.44)	0.94 (0.66–1.31)
EDHS-2011			1.07 (0.89–1.29)	0.89 (0.68–1.15)
EDHS-2016			Ref.	Ref.
**Random Effect**				
Variance (SE)	0.2438 (0.0053)***	0.2378 (0.0123)**	0.2673 (0.0054)***	0.2384 (0.0122)**
ICC (95%CI)	6.90 (3.99–11.66)	6.74 (2.87–15.00)	7.51 (4.44–12.44)	6.75 (2.89–14.96)
**Model fit statistics**				
AIC	5450.24	3284.06	5415.43	3287.58
BIC	5466.00	3574.86	5470.59	3614.74
LL	-2723.12	-1602.03	-2700.72	-1598.79
Deviance	5,446.24	3,204.06	5,401.44	3,197.59

*p<0.05

**p<0.001

***p<0.0001

## Discussion

Ethiopia is currently known to be the most food-insecure country in sub-Saharan Africa, where childhood undernutrition remains endemic. Concomitantly, there is evidence of high burden of childhood undernutrition and anaemia. Current global nutritional transitions are likely to aggravate the situation and foster a new dimension of the problem which include underweight, hidden hunger (micronutrient deficiencies) and overweight collectively referred to as the "triple burden of malnutrition" (TBM). While attention is increasingly being paid to childhood undernutrition in Ethiopia, a current surge of TBM has received less attention. We, therefore, aimed to examine the prevalence and identify the factors associated with TBM among mother–child pairs. Overall, the prevalence of TBM was 2.6% among mother-child pairs in Ethiopia. Factors associated with increased odds of TBM included: older child age (aged 12–23 months and aged 24–35 months), children reported to be smaller than normal at birth, children who had a fever, short maternal height, time to get drinking water, and urban residence. On the other hand, lower odds of TBM were identified with the female sex, mothers having a non-agriculture occupation, children born to mothers who attended antenatal care (ANC), and households within the relatively rich wealth category.

The present finding of the prevalence of TBM was lower than a study finding based on the National Family Health Survey 2015–16 in India (5.7%) [14], 4.9% in Bangladesh [34], and 7% in Nepal [3]. The low prevalence of maternal overweight/obesity in Ethiopia may be the reason for the observed disparity. In Ethiopia, 8% of women between the ages of 15 and 49 are overweight or obese, according to the EDHS-2016 data [12], while the prevalence of overweight or obese among women between the ages of 15 and 49 was found to be 22% in Nepal in the same year [35]. It is noteworthy that socio-demographic, economic differences, and other determinants towards addressing malnutrition in all its forms, could account for variations in the prevalence of TBM. However, our finding demonstrated that the TBM was higher in Ethiopia than data from 32 countries in SSA 1% [4]. A close examination of the TBM trends reveals that it was 3.8% in the EDHS-2005, 3.12% in the EDHS-2011, and 3.0% in the EDHS-2016, and that there was no significant decrease in the prevalence during these years as the confidence intervals overlapped. Several factors seem to have contributed to the prevalence of TBM in Ethiopia. These include: rapid urbanization, economic growth, a shift in disease burden, and nutritional transition. It is reasonable to argue that the observed high burden of TBM has been exacerbated by the high levels of stunting and anemia in the country.

Our study shows that TBM was less likely to occur among female children. This result is consistent with that of Ahinkorah et al., [4] that reported TBM to be less likely to occur among female compared to male children. Several studies have shown that being a female child is strongly tied to lower odds of undernourishment in Ethiopia [36–41].

This study shows that children who were perceived to be smaller sized than average at birth were strongly associated with higher odds of TBM. This result is consistent with studies from SSA [4], India [14], Ethiopia [42], and Bangladesh [43]. This result is not unexpected considering that low birth weight is highly correlated with child undernutrition and anemia [44–47] and multiple concurrent forms of undernutrition [42,43]. The observed relationship between TBM and perceived birth size could have been attributed to biological and maternal nutrition status during pregnancy.

In this study, a higher child age is significantly associated with higher risks of TBM and consistent with a previous study by Sunuwar et al. conducted in Nepal, that utilized the Nepal Demographic and Health Survey (NDHS) 2016 [3], where children aged 12–35 months were more likely to experience TBM. Prior evidence in Ethiopia showed that childhood anemia and undernutrition are more severe in older children [36,40,45,48], explaining the observed higher odds of TBM among children aged 12 or 35 months. Children born to mothers with short height had higher odds of experiencing TBM compared to those born to mothers who had normal height. These results are congruent with reports from elsewhere and in Ethiopia which discussed the association between maternal statures and DBM, such as studies from Indonesia and Bangladesh [15], Nepal [3], Ethiopia [49], Guatemala [50], and Brazil [51]. Some of the pathways through which the maternal stature is associated with child malnutrition have been described in detail elsewhere [49,52–54]. For instance, studies reported that Body Mass Index (BMI) gain was higher in short-statured mother than those with normal height [55] and women of short stature are more likely to have undernourished children [56,57].

Maternal occupation and household wealth status were drivers associated with the household-level TBM. Children of mothers who worked in non-agriculture occupation were less likely to report TBM compared to children of mothers not working in paid jobs. The role of maternal employment on poor child development have been previously reported [58,59]. For example, a recent study conducted by Bliznashka et al. (2023) [60] on the associations between parental employment (comparing agricultural and non-agricultural employment) and child development found that parental agricultural employment was associated with poorer child development. To our knowledge, no studies have examined the association between maternal occupation and TBM. Hence, more research is needed to unpack these associations.

Compared to children from poor households, children from rich households had significantly decreased odds of TBM. A lower rate of TBM among richer wealth could reflect that a significant number of children aged 6–59 months were found to be anaemic in those in the poorest and poorer wealth category in Ethiopia [48]. In addition, a high prevalence of TBM was found in households with poor households than those in rich wealth category (4.27 vs 1.63, p<0.001). This finding might also be because mother-children from higher wealth quintiles are more likely to receive a balanced diet that includes adequate macronutrients and micronutrients.

The odds of TBM were significantly lower among children born to mothers who attended ANC compared to those born to mothers who did not attend ANC. This is in agreement with a prior study done in SSA [4]. This finding might be attributed to mothers receiving ANC may be exposed to various health education programs and supplementation with folic acid, which supports maternal health and meeting the nutritional needs of the child, which may help to explain this finding. In addition, the odds of TBM were higher among those children who had a fever in the last fifty days prior to the survey. Even though the cross-sectional data type prevents us from seeing the association clearly, the underlying cause of fever could be different. It can be caused by systemic infections in the body, which can affect the hemoglobin level in the blood and cause anemia, resulting in TBM.

Households with children who spent time fetching water were at higher odds of experiencing TBM than children born to mothers whose households have water access on their premises. One possible explanation for this finding is that time to drinking water sources may be a proxy indicator for poor health and, in many cases, associated with childhood undernutrition [40,61–64]. Lack of access to drinking water in conjunction with other factors may result in TBM at the household level. Therefore, the current findings emphasize the need to invest in a clean, safe, and accessible water supply in Ethiopia. Furthermore, TBM was more likely to occur among urban residents compared to rural dwellers. Despite the limited evidence on TBM available, place of residence on TBM is a critical factor, with studies reporting similar results [3,4,14].

Although this study contributes to the scant literature on TBM in Ethiopia, it has limitations. Due to the cross‐sectional study design, cause‐effect relationships could not be established in the current study. Due to a lack of relevant data in EDHS, the potential confounding factors, such as household food insecurity were not included in the study, which could have influenced the observed TBM prevalence. Some variables included in this study were subject to social desirability and recall bias. Finally, the pooling of the data of may be affected by heterogeneity across the three waves.

## Conclusions

The overall prevalence of TBM was 2.6% in Ethiopia. TBM was less likely to occur among female children compared to males. Children from others with non-agriculture occupations, mothers who attended antenatal care, and those who were from households within the relatively rich wealth category had lower odds of experiencing TBM compared to their counterparts. Children aged 12–23 months and aged 24–35 months, children perceived as smaller size than average at birth, those who had fever in the last fifty days prior to the survey, children born to mothers with short height, and those living in an urban areas had higher odds of experiencing TBM. Addressing the TBM through double-duty actions will be of critical importance in achieving malnutrition in all its forms in Ethiopia and the Sustainable Development Goals 2.

## References

[1] Kosaka S, Umezaki M. A systematic review of the prevalence and predictors of the double burden of malnutrition within households. Br J Nutr. 2017;117(8):1118–27. doi: 10.1017/S0007114517000812 28514987

[2] Davis JN, Oaks BM, Engle-Stone R. The Double Burden of Malnutrition: A Systematic Review of Operational Definitions. Curr Dev Nutr. 2020;4(9):nzaa127. Available from: doi: 10.1093/cdn/nzaa127 32885132 PMC7456307

[3] Sunuwar DR, Singh DR, Pradhan PMS. Prevalence and factors associated with double and triple burden of malnutrition among mothers and children in Nepal: evidence from 2016 Nepal demographic and health survey. BMC Public Health. 2020;20(1):1–11. Available from: https://doi/10.1186/s12889-020-8356-y 32223749 10.1186/s12889-020-8356-y PMC7104542

[4] Ahinkorah BO, Amadu I, Seidu AA, Okyere J, Duku E, Hagan JE, et al. Prevalence and Factors Associated with the Triple Burden of Malnutrition among Mother-Child Pairs in Sub-Saharan Africa. Nutrients. 2021;13(6):2050. Available from: doi: 10.3390/nu13062050 34203986 PMC8232587

[5] Mamun S, Mascie-Taylor CGN. Double Burden of Malnutrition (DBM) and Anaemia under the Same Roof: A Bangladesh Perspective. Med Sci. 2019;7(2):20. Available from: doi: 10.3390/medsci7020020 30696099 PMC6409552

[6] Tarekegn BT, Assimamaw NT, Atalell KA, Kassa SF, Muhye AB, Techane MA, et al. Prevalence and associated factors of double and triple burden of malnutrition among child-mother pairs in Ethiopia: Spatial and survey regression analysis. BMC Nutr. 2022;8(1):34. Available from: doi: 10.1186/s40795-022-00528-5 35449087 PMC9027462

[7] FAO, UNICEF, WFP and WHO. The State of Food Security and Nutrition in the World 2020. Transforming food systems for affordable healthy diets. FAO, Rome. 2000. Available from: https://www.fao.org/3/ca9692en/ca9692en.pdf

[8] UNICEF, WHO and the World Bank Group. Levels and trends in child malnutrition: UNICEF/WHO/The World Bank Group joint child malnutrition estimates: key findings of the 2021. [cited 2023 Nov 16]. Available from: https://www.who.int/publications-detail-redirect/9789240025257

[9] Takele BA, Gezie LD, Alamneh TS. Pooled prevalence of stunting and associated factors among children aged 6–59 months in Sub-Saharan Africa countries: A Bayesian multilevel approach. PLOS ONE. 2022;17(10):e0275889. Available from: doi: 10.1371/journal.pone.0275889 36228030 PMC9560624

[10] Anaemia in women and children. [cited 2023 Jun 9]. Available from: https://www.who.int/data/gho/data/themes/topics/anaemia_in_women_and_children

[11] EPHI and ICF. EPHI ICF. Ethiopia MiniDemographic and Health Survey 2019: Key indicators. Rockville, Maryland, USA: EPHI and ICF. 2019.

[12] EDHS. Central Statistical Agency (CSA) [Ethiopia] and ICF. 2016. Ethiopia Demographic and Health Survey 2016. Addis Ababa, Ethiopia, and Rockville, Maryland, USA: CSA and ICF. 2016.

[13] WHO. Haemoglobin concentrations for the diagnosis of anaemia and assessment of severity. Vitamin and Mineral Nutrition Information System. Available from: http://www.who.int/vmnis/indicators/haemoglobin.pdf

[14] Kumar P, Chauhan S, Patel R, Srivastava S, Bansod DW. Prevalence and factors associated with triple burden of malnutrition among mother-child pairs in India: a study based on National Family Health Survey 2015–16. BMC Public Health. 2021;21(1):391. Available from: doi: 10.1186/s12889-021-10411-w 33622303 PMC7901069

[15] Oddo VM, Rah JH, Semba RD, Sun K, Akhter N, Sari M, et al. Predictors of maternal and child double burden of malnutrition in rural Indonesia and Bangladesh. Am J Clin Nutr. 2012;95(4):951–8. Available from: doi: 10.3945/ajcn.111.026070 22357721

[16] Bliznashka L, Blakstad MM, Berhane Y, Tadesse AW, Assefa N, Danaei G, et al. Household-level double burden of malnutrition in Ethiopia: a comparison of Addis Ababa and the rural district of Kersa. Public Health Nutr. 2021;24(18):6354–68. Available from: doi: 10.1017/S1368980021003700 34446127 PMC11148622

[17] Ministry of Health (MoH) Ethiopia. Seqota Declaration. [cited 2023 Nov 15]. Available from: https://www.moh.gov.et/site/am/node/170

[18] Seife Ayele EAZ, Nisbett N. Multi-Sectoral Nutrition Policy and Programme Design, Coordination and Implementation in Ethiopia. IDS; 2020.

[19] Roba AA, Assefa N, Dessie Y, Tolera A, Teji K, Elena H, et al. Prevalence and determinants of concurrent wasting and stunting and other indicators of malnutrition among children 6–59 months old in Kersa, Ethiopia. Matern Child Nutr. 2021;17(3):e13172. Available from: doi: 10.1111/mcn.13172 33728748 PMC8189198

[20] Pradeilles R, Irache A, Norris T, Chitekwe S, Laillou A, Baye K. Magnitude, trends and drivers of the coexistence of maternal overweight/obesity and childhood undernutrition in Ethiopia: Evidence from Demographic and Health Surveys (2005–2016). Matern Child Nutr. 2022; e13372 Available from: doi: 10.1111/mcn.13372 35615766 PMC11258774

[21] Sebsbie A, Minda A, Ahmed S. Co-existence of overweight/obesity and stunting: it’s prevalence and associated factors among under—five children in Addis Ababa, Ethiopia. BMC Pediatr. 2022;22(1):377. Available from: 10.1186/s12887-022-03445-5 35764944 PMC9241306

[22] Amare HH, Lindtjorn B. Concurrent anemia and stunting among schoolchildren in Wonago district in southern Ethiopia: a cross-sectional multilevel analysis. PeerJ. 2021;9. Available from: 10.7717/peerj.11158 PMC8106909 33996276

[23] Farah AM, Nour TY, Endris BS, Gebreyesus SH. Concurrence of stunting and overweight/obesity among children: Evidence from Ethiopia. PLOS ONE. 2021;16(1):e0245456. Available from: doi: 10.1371/journal.pone.0245456 33449970 PMC7810347

[24] Eshete T, Kumera G, Bazezew Y, Marie T, Alemu S, Shiferaw K. The coexistence of maternal overweight or obesity and child stunting in low-income country: Further data analysis of the 2016 Ethiopia demographic health survey (EDHS). Sci Afr. 2020;9:e00524. Available from: 10.1016/j.sciaf.2020.e00524

[25] EDHS. Central Statistical Agency [Ethiopia] and ORC Macro. Ethiopia Demographic and Health Survey 2005. Central Statistical Agency/Ethiopia and ORC Macro; 2006. 2005;

[26] EDHS. Central Statistical Agency [Ethiopia] and ICF International. Ethiopia Demographic and Health Survey 2011. Central Statistical Agency and ICF International; 2012. 2011;

[27] WHO Child Growth Standards, 2006. Length/Height-for-Age, Weight-for-Age, Weight-for-Length, Weight-for-Height and Body Mass Index-for-Age. Available from: https://www.who.int/publications/i/item/924154693X

[28] World Health Organization. Diet, nutrition, and the prevention of chronic diseases: report of a joint WHO/FAO expert consultation. Geneva: World Health Organization. 2003.

[29] World Health Organization. 2015. The Global Prevalence of Anemia in 2011. Geneva: World Health Organization.

[30] Tran TD, Biggs BA, Holton S, Nguyen HTM, Hanieh S, Fisher J. Co-morbid anaemia and stunting among children of pre-school age in low- and middle-income countries: a syndemic. Public Health Nutr. 2019;22(1):35–43. Available from: doi: 10.1017/S136898001800232X 30246676 PMC10260636

[31] Mohammed SH, Larijani B, Esmaillzadeh A. Concurrent anemia and stunting in young children: prevalence, dietary and non-dietary associated factors. Nutr J. 2019;18(1):10. Available from: doi: 10.1186/s12937-019-0436-4 30791904 PMC6385383

[32] Vatcheva KP, Lee M, McCormick JB, Rahbar MH. Multicollinearity in Regression Analyses Conducted in Epidemiologic Studies. Epidemiol Open Access. 2016. 6(2): 227. doi: 10.4172/2161-1165.1000227 27274911 PMC4888898

[33] Merlo J. A brief conceptual tutorial of multilevel analysis in social epidemiology: using measures of clustering in multilevel logistic regression to investigate contextual phenomena. J Epidemiol Community Health. 2006;60(4):290–7. Available from: doi: 10.1136/jech.2004.029454 16537344 PMC2566165

[34] Das S, Fahim SM, Islam MS, Biswas T, Mahfuz M, Ahmed T. Prevalence and sociodemographic determinants of household-level double burden of malnutrition in Bangladesh. Public Health Nutr. 2019;22(8):1425–32. Available from: doi: 10.1017/S1368980018003580 30612592 PMC10260912

[35] Rana K, Ghimire P, Chimoriya R, Chimoriya R. Trends in the Prevalence of Overweight and Obesity and Associated Socioeconomic and Household Environmental Factors among Women in Nepal: Findings from the Nepal Demographic and Health Surveys. Obesities. 2021;1(2):113–35. Available from: 10.3390/Obesities1020011

[36] Asfaw M, Wondaferash M, Taha M, Dube L. Prevalence of undernutrition and associated factors among children aged between six to fifty nine months in Bule Hora district, South Ethiopia. BMC Public Health. 2015;15(1):41. Available from: doi: 10.1186/s12889-015-1370-9 25636688 PMC4314803

[37] Roba AA, Assefa N, Dessie Y, Tolera A, Teji K, Elena H, et al. Prevalence and determinants of concurrent wasting and stunting and other indicators of malnutrition among children 6–59 months old in Kersa, Ethiopia. Matern Child Nutr. 2021;17(3):e13172. Available from: 10.1111/mcn.13172 33728748 PMC8189198

[38] Tesfaw LM, Woya AA. Potential mediators of the link between wealth index and anthropometric indices of under-five children in Ethiopia. Front Public Health. 2022;10:981484. Available from: doi: 10.3389/fpubh.2022.981484 36311561 PMC9606741

[39] Muche A, Dewau R. Severe stunting and its associated factors among children aged 6–59 months in Ethiopia; multilevel ordinal logistic regression model. Ital J Pediatr. 2021;47(1):161. Available from: 10.1186/s13052-021-01110-8 34311750 PMC8314542

[40] Sahiledengle B, Mwanri L, Petrucka P, Kumie A, Beressa G, Atlaw D, et al. Determinants of undernutrition among young children in Ethiopia. Sci Rep. 2022;12(1):20945. Available from: doi: 10.1038/s41598-022-25160-y 36470914 PMC9722653

[41] Gebreegziabher T, Regassa N. Ethiopia’s high childhood undernutrition explained: analysis of the prevalence and key correlates based on recent nationally representative data. Public Health Nutr. 2019;22(11):2099–109. Available from: doi: 10.1017/S1368980019000569 30894232 PMC10260527

[42] Sahiledengle B, Agho KE, Petrucka P, Kumie A, Beressa G, Atlaw D, et al. Concurrent wasting and stunting among under‐five children in the context of Ethiopia: A generalised mixed‐effects modelling. Matern Child Nutr. 2023;19: e13483. Available from: doi: 10.1111/mcn.13483 36757269 PMC10019057

[43] Chowdhury MRK, Khan HTA, Rashid M, Kabir R, Islam S, Shariful Islam M, et al. Differences in risk factors associated with single and multiple concurrent forms of undernutrition (stunting, wasting or underweight) among children under 5 in Bangladesh: a nationally representative cross-sectional study. BMJ Open. 2021;11(12):e052814. Available from: doi: 10.1136/bmjopen-2021-052814 34903543 PMC8672009

[44] Ayelign A, Zerfu T. Household, dietary and healthcare factors predicting childhood stunting in Ethiopia. Heliyon. 2021;7(4):e06733. Available from: doi: 10.1016/j.heliyon.2021.e06733 33912713 PMC8066354

[45] Fantay Gebru K, Mekonnen Haileselassie W, Haftom Temesgen A, Oumer Seid A, Afework Mulugeta B. Determinants of stunting among under-five children in Ethiopia: a multilevel mixed-effects analysis of 2016 Ethiopian demographic and health survey data. BMC Pediatr. 2019;19(1):176. Available from: doi: 10.1186/s12887-019-1545-0 31153381 PMC6544992

[46] Akombi BJ, Agho KE, Hall JJ, Wali N, Renzaho AMN, Merom D. Stunting, Wasting and Underweight in Sub-Saharan Africa: A Systematic Review. Int J Environ Res Public Health. 2017;14(8):863. Available from: doi: 10.3390/ijerph14080863 28788108 PMC5580567

[47] Figueiredo ACMG Gomes-Filho IS, Batista JET Orrico GS, Porto ECL Cruz Pimenta RM, et al. Maternal anemia and birth weight: A prospective cohort study. PLOS ONE. 2019 Mar 18;14(3):e0212817. Available from: doi: 10.1371/journal.pone.0212817 30884493 PMC6422668

[48] Anteneh ZA, Van Geertruyden JP. Spatial variations and determinants of anemia among under-five children in Ethiopia, EDHS 2005–2016. PLOS ONE. 2021;16(4):e0249412. Available from: doi: 10.1371/journal.pone.0249412 33793640 PMC8016260

[49] Sahiledengle B, Mwanri L, Agho KE. Association between maternal stature and household-level double burden of malnutrition: findings from a comprehensive analysis of Ethiopian Demographic and Health Survey. J Health Popul Nutr. 2023;42(1):7. Available from: doi: 10.1186/s41043-023-00347-9 36691083 PMC9872360

[50] Doak CM, Campos Ponce M, Vossenaar M, Solomons NW. The stunted child with an overweight mother as a growing public health concern in resource-poor environments: a case study from Guatemala. Ann Hum Biol. 2016;43(2):122–30. Available from: doi: 10.3109/03014460.2015.1136356 26863530

[51] Géa-Horta T, Silva R de CR, Fiaccone RL, Barreto ML, Velásquez-Meléndez G. Factors associated with nutritional outcomes in the mother–child dyad: a population-based cross-sectional study. Public Health Nutr. 2016;19(15):2725–33. Available from: doi: 10.1017/S136898001600080X 27121979 PMC10271133

[52] Félix-Beltrán L, Macinko J, Kuhn R. Maternal height and double-burden of malnutrition households in Mexico: stunted children with overweight or obese mothers. Public Health Nutr. 2021;24(1):106–16. Available from: doi: 10.1017/S136898002000292X 32867877 PMC10049080

[53] Addo OY, Stein AD, Fall CH, Gigante DP, Guntupalli AM, Horta BL, et al. Maternal height and child growth patterns. J Pediatr. 2013;163(2):549–54. Available from: doi: 10.1016/j.jpeds.2013.02.002 23477997 PMC3711792

[54] Karlsson O, Kim R, Bogin B, Subramanian S. Maternal Height-standardized Prevalence of Stunting in 67 Low- and Middle-income Countries. J Epidemiol. 2022;32(7):337–44. Available from: doi: 10.2188/jea.JE20200537 33612705 PMC9189321

[55] Sichieri R, Silva CVC, Moura AS. Combined effect of short stature and socioeconomic status on body mass index and weight gain during reproductive age in Brazilian women. Braz J Med Biol Res. 2003;36(10):1319–25. Available from: doi: 10.1590/s0100-879x2003001000007 14502363

[56] Gupta A, Cleland J, Sekher TV. Effects of parental stature on child stunting in India. J Biosoc Sci. 2022;54(4):605–16. Available from: doi: 10.1017/S0021932021000304 34275505

[57] Ferreira HS, Moura FA, Cabral CR, Florêncio TMMT, Vieira RC, de Assunção ML. Short stature of mothers from an area endemic for undernutrition is associated with obesity, hypertension and stunted children: a population-based study in the semi-arid region of Alagoas, Northeast Brazil. Br J Nutr. 2009;101(8):1239–45. Available from: 10.1017/S0007114508059357 19017417

[58] Nankinga O, Kwagala B, Walakira EJ. Maternal employment and child nutritional status in Uganda. PLOS ONE. 2019;14(12):e0226720. Available from: doi: 10.1371/journal.pone.0226720 31856209 PMC6922416

[59] Shuhaimi F, Muniandy ND. The Association of Maternal Employment Status on Nutritional Status among Children in Selected Kindergartens in Selangor, Malaysia. Asian J Clin Nutr. 2012;4(2):53–66. Available from: 10.3923/ajcn.2012.53.66

[60] Bliznashka L, Jeong J, Jaacks LM. Maternal and paternal employment in agriculture and early childhood development: A cross-sectional analysis of Demographic and Health Survey data. PLOS Glob Public Health. 2023;3(1):e0001116. Available from: doi: 10.1371/journal.pgph.0001116 36962809 PMC10021554

[61] Ademas A, Adane M, Keleb A, Berihun G, Tesfaw G. Water, sanitation, and hygiene as a priority intervention for stunting in under-five children in northwest Ethiopia: a community-based cross-sectional study. Ital J Pediatr. 2021;47(1):174. Available from: doi: 10.1186/s13052-021-01128-y 34429146 PMC8385795

[62] Arnold BF, Null C, Luby SP, Unicomb L, Stewart CP, Dewey KG, et al. Cluster-randomised controlled trials of individual and combined water, sanitation, hygiene and nutritional interventions in rural Bangladesh and Kenya: the WASH Benefits study design and rationale. BMJ Open. 2013;3(8):e003476. Available from: doi: 10.1136/bmjopen-2013-003476 23996605 PMC3758977

[63] Baldi AJ, Clucas D, Pasricha SR. Anemia and water, sanitation, and hygiene (WASH)—is there really a link? Am J Clin Nutr. 2020;112(5):1145–6. Available from: doi: 10.1093/ajcn/nqaa213 32692804

[64] Bekele T, Rahman B, Rawstorne P. The effect of access to water, sanitation and handwashing facilities on child growth indicators: Evidence from the Ethiopia Demographic and Health Survey 2016. PLOS ONE. 2020;15(9):e0239313. Available from: doi: 10.1371/journal.pone.0239313 32960921 PMC7508389

